# The Prize Plans for the King's Sanatorium

**Published:** 1903-01-10

**Authors:** 


### The Hospital.

A Journal of

The Medical Sciences and Hospital Administration.

Vol. XXXIII.—No. 850.] Edited by Sir Henry Burdett, K.C.B., and Solomon C. Smith, M.D., M.R.C.P. [January 10, 1903.

### The Prize Plans for the King's Sanatorium.

The publication of the three essays on the Sanatorium Treatment of Tuberculosis which were successful in obtaining the prizes offered by direction of his Majesty, draws attention afresh to the many problems involved in any serious attempt to lessen the mortality caused by phthisis in this country. To the essays themselves we must accord the highest praise. Although somewhat different views in regard to certain points were adopted by the several authors, each essay shows signs not only of much thought and careful preparation, but of an intimate acquaintance with the whole subject, and each of them well deserves the position it has attained. No one can doubt, however, as it seems to us, that the one submitted by Dr. Arthur Latham, with whom is associated as architect Mr. A. William West, stands *facile princeps*, and that it has thoroughly well earned the premium which it has obtained.

Into the details of the essay and plans we will enter more fully on a future occasion; our present interest lies rather in the light thrown by them upon the general question. Dr. Latham fully accepts the dictum of Brehmer, who said that "anything which protects one man from falling ill must be able, if properly employed, to cure another of the same disease," and he has endeavoured, in arranging his buildings and his régime, to put this saying into practice—placing his patients amid conditions which are known to be inimical to the spread of the disease, and eliminating from their surroundings all those influences which are recognised as conducive to the onset and the progress of the malady. Tuberculosis truly has its origin in an infective micro-organism. But for all that it is on the one hand a filth disease, arising from the breathing of infected dust and the eating of infected food, while on the other it is a mode of death in conditions of debility induced by insanitary living. Thus it happens that for cure as for prevention the surroundings must be made absolutely cleanly, while the individual's power of resistance must be so increased, by appropriate diet and régime, that his frame shall become practically immune to the attacks of the bacillus.

That all this can be effected by sanatorium treatment we have no doubt; in fact, that it has been done in numerous cases we know. But we know also, and this essay does but confirm and emphasise the point, that such a process of cure is a very expensive affair; and when we come to examine the elaborate and costly provision made by the essayists for the comparatively small number of patients who are to be treated in the proposed sanatorium, we cannot but ask, How is all this to be provided for the vast array of consumptives who are waiting to be dealt with? Unless sanatorium treatment be made available, not for the lucky few alone, but for the whole crowd of sufferers, the provision of such treatment touches the very fringe only of the great tuberculosis problem, a problem which obviously must be dealt with by prevention rather than by cure.

The gravity of the whole question, especially the difficulty of dealing with consumption among the poor, is strongly brought home to us by what is said about the care and the precautions required to prevent relapse among those who are recovering and recurrence among those who are even convalescent; and when we compare the elaborate precautions which are considered desirable, if not necessary, while the patients remain in the sanatorium, with the penurious surroundings to which these convalescents must return when the disease is at last "cured" or arrested, the contrast is horrible. It is this that snatches away, as from our very lips, the satisfaction felt at the wonders that are worked by sanatorium treatment.

The noble sanatorium which at the King's bidding will shortly arise will without doubt be an untold blessing to a large number of suffering individuals, and in proportion as cures are effected will tend to diminish the number of foci of infection. But from a public health point of view it will be chiefly as an example that it will do good—an example proving directly how consumption may be cured by attention to hygienic surroundings, and proving by inference that it is to the lack of such sanitary surroundings that the origin of the disease is to be traced. The prevalence of tuberculosis is to be diminished by stringent laws and their firm enforcement in regard to overcrowding, by the intrusion of the municipal scrubber and disinfector wherever illness is known to prevail, by the maintenance of a high standard of building construction, by the enforcement of cleanly living, even by aid of law if necessary, by carefully-exercised supervision over food supplies, by strict regulation of workshops, by the suppression of evil habits, such as that of spitting, by the force of public opinion, and, lastly, by the gentle influence of the health missioner preaching in the homes of the people the doctrine of pure air, sweetness, and light. Meanwhile, the King, and with him Sir Ernest Cassel, will be blessed in many a home for the restoration of the breadwinner to the family circle.

